# Supplementary material for: Methylation reprogramming associated with aggressive prostate cancer and ancestral disparities
Source: Mol Syst Biol. 2025 Oct 7;21(12):1676–701. doi: 10.1038/s44320-025-00153-x (PMC12673094; doi:10.1038/s44320-025-00153-x)
Supplement: Supplementary file 1 — Appendix [file 44320_2025_153_MOESM1_ESM.pdf]

Appendix

Methylation reprogramming associated with aggressive prostate cancer and ancestral disparities

Jenna Craddock, Pavlo Lutsik, Pamela X.Y. Soh, Melanie Louw, Md. Mehedi Hasan, Sean M. Patrick, Shingai B.A. Mutambirwa, Phillip D. Stricker, Hagen E.A. Förtsch, HEROIC PCaPH Africa1K Consortium, M.S. Riana Bornman, Clarissa Gerhäuser, and Vanessa M. Hayes

Table of Contents

Appendix Figures 3

Appendix Figure S1 - EPICv1 and EPICv2 cross-platform reproducibility between matched African sample pairs. 3

Appendix Figure S2 - African prostate tumour misclassification. 4

Appendix Figure S3 - Principal component analysis (PCA) of African *versus* non-African prostate tumours. 5

Appendix Figure S4 - Principal component analysis (PCA) of African and non-African prostate tumours annotated by tumour purity. 6

Appendix Figure S5 - Global DNA methylation across repetitive elements in African *versus* non-African prostate tumours. 7

Appendix Figure S6 - Ancestry-associated hyper- and hypomethylated differentially methylated positions (DMPs) and regions (DMRs) across genomic features. 8

Appendix Figure S7 - Methylation at top 10 ancestry-associated DMPs by tumour purity and ancestry. 9

Appendix Figure S8 - Methylation at top 10 DMPs across tumour purity quartiles by ancestry. 11

Appendix Figure S9 - Methylation at top 10 ancestry-associated DMRs by tumour purity and ancestry. 13

Appendix Figure S10 - Methylation at top 10 DMRs across tumour purity quartiles by ancestry. 15

Appendix Figure S11 - Principal component analysis (PCA) of African *versus* non-African prostate tumours in the ancestry-associated validation cohort. 17

Appendix Figure S12 - DMP cluster analysis heatmap by ancestry, geography, ISUP grade group and chromatin state in the ancestry-associated validation cohort. 18

Appendix Figure S13 - Volcano plot for differentially methylated positions (DMPs) between African and non-African prostate tumour-derived samples in the ancestry-associated validation cohort. 19
Appendix Figure S14 - Ancestry-associated DMP and DMR enrichment across gene and CpG island regions, and chromatin state contexts in the validation cohort........ 20
Appendix Figure S15 - Principal component analysis (PCA) of African prostate tumour *versus* normal tissue. 21
Appendix Figure S16 - Principal component analysis (PCA) of African prostate tumour *versus* normal tissue by cell types. 22
Appendix Figure S17 - Global DNA methylation across Alu repetitive elements in African prostate tumour *versus* normal tissue. 23
Appendix Figure S18 - Tumour-associated hyper- and hypomethylated differentially methylated positions (DMPs) and regions (DMRs) across genomic features and detailed chromatin states. 24
Appendix Figure S19 - Principal component analysis (PCA) of African prostate tumour *versus* normal tissue in the tumour-associated validation cohort. 25
Appendix Figure S20 - DMP cluster analysis heatmap by cancer status, ISUP grade group and chromatin state in the tumour-associated validation cohort. 26
Appendix Figure S21 - Global DNA methylation across repetitive elements and CpG island-related regions in African prostate tumour *versus* normal tissue in the tumour-associated validation cohort. 27
Appendix Figure S22 - Volcano plot for differentially methylated positions (DMPs) between African prostate tumour and normal tissue in the tumour-associated validation cohort. 28
Appendix Figure S23 - Tumour-associated DMP and DMR enrichment across gene and CpG island regions, and chromatin state contexts in the validation cohort........ 29

Appendix Figures

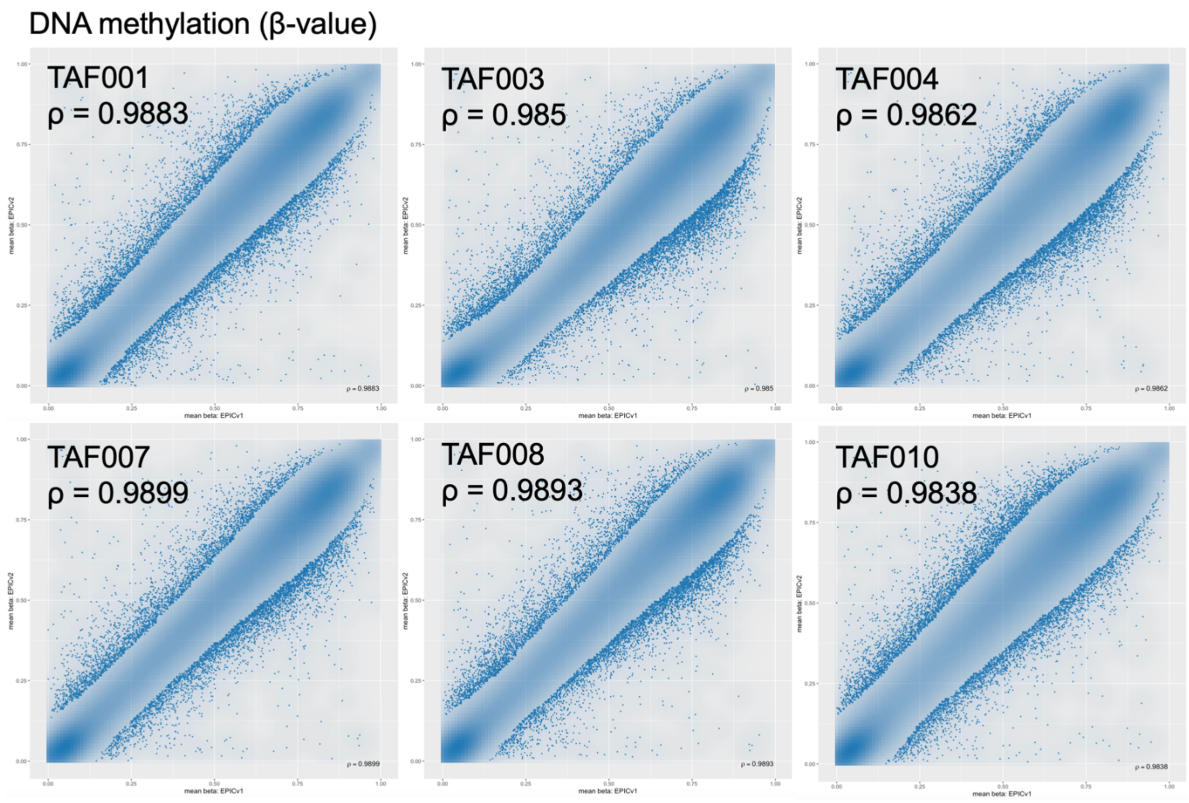

Appendix Figure S1 - EPICv1 and EPICv2 cross-platform reproducibility between matched African sample pairs. Scatterplots showing the correlation between methylation measurements from EPICv1 and EPICv2 for six African replicate pairs: TAF001, TAF003, TAF004, TAF007, TAF008 and TAF010.

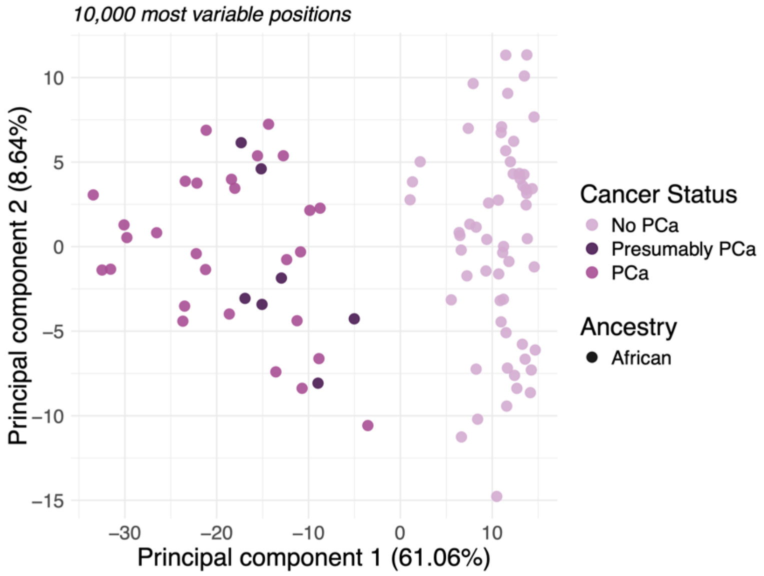

Appendix Figure S2 - African prostate tumour misclassification. Principal component analysis plot of 93 African EPICv2 samples across the 10,000 most variable CpG positions, indicating samples histologically classified as non-tumour, but displaying DNA methylation profiles consistent with prostate tumour samples (i.e. “Presumably prostate cancer (PCa)”). African samples are annotated according to cancer status: those with PCa (n = 28), those without PCa (n = 58), and those presumably with PCa (n = 7).

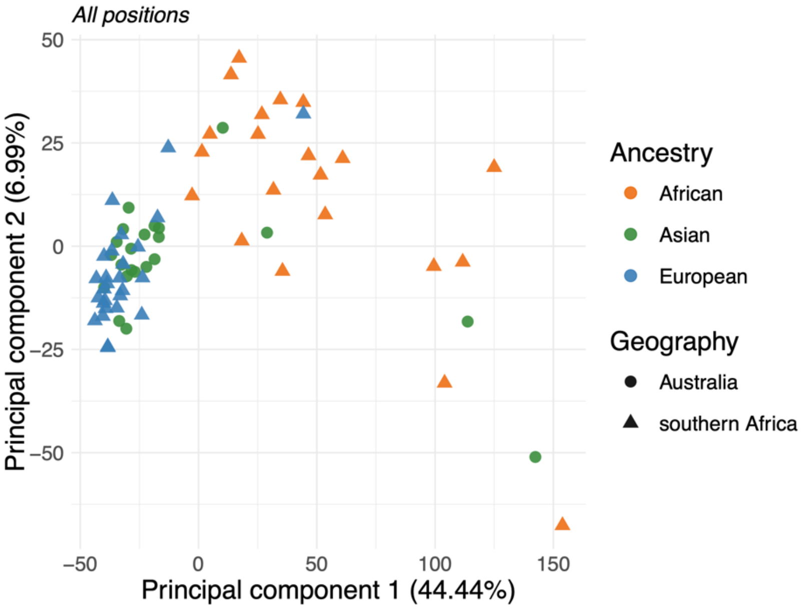

Appendix Figure S3 - Principal component analysis (PCA) of African *versus* non-African prostate tumours. PCA plot of 70 EPICv1 samples across all positions. Samples are annotated according to geography: Australia (*n* = 22) and South Africa (*n* = 48); and ancestry: African (*n* = 21), Asian (*n* = 22) and European (*n* = 27).

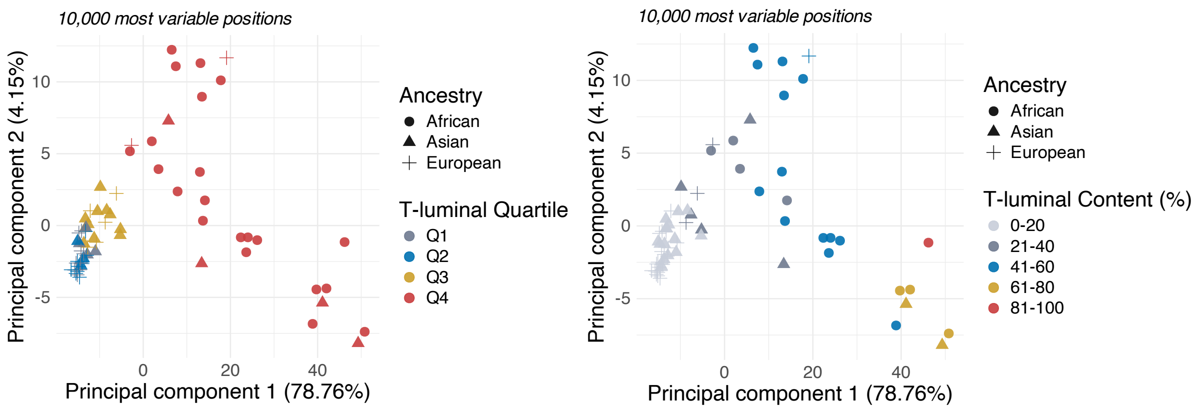

Appendix Figure S4 - Principal component analysis (PCA) of African and non-African prostate tumours annotated by tumour purity. PCA plot of 70 EPICv1 samples across the 10,000 most variable positions indicating (*left*) T-luminal quartile and (*right*) estimated T-luminal content. Samples are annotated according to ancestry: African (n = 21), Asian (n = 22) and European (n = 27); T-luminal quartile: Q1 (n = 14), Q2 (n = 13), Q3 (n = 16) and Q4 (n = 27); and estimated T-luminal percentage: 0-20 (n = 38), 21-40 (n = 12), 41-60 (n = 14), 61-80 (n = 5) and 81-100 (n = 1).

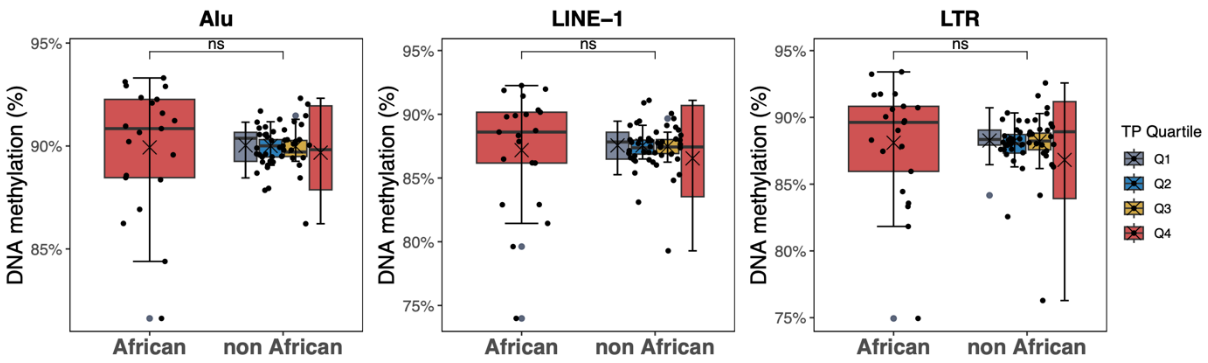

Appendix Figure S5 - Global DNA methylation across repetitive elements in African *versus* non-African prostate tumours. Boxplots showing covariate-adjusted global DNA methylation levels stratified by ancestry (African vs. non-African) and tumour purity (TP) quartile (Q1-Q4) across three classes of repetitive elements: Alu, LINE-1 and long tandem repeats (LTRs). Each point represents the median methylation value for an individual tumour sample. Group means are indicated by an “X”.
Abbreviations: *ns*, not significant.

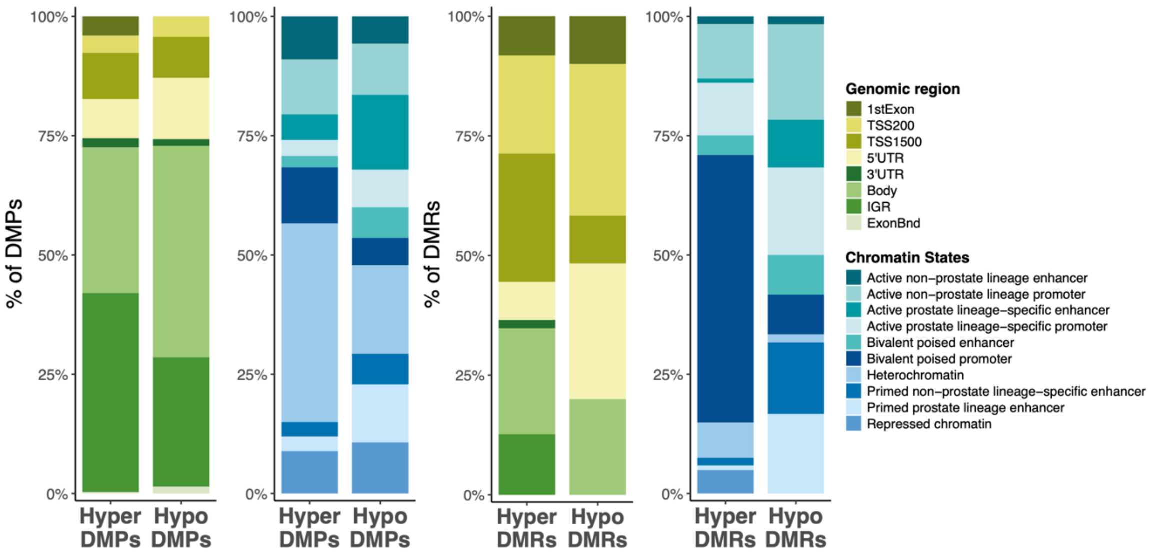

Appendix Figure S6 - Ancestry-associated hyper- and hypomethylated differentially methylated positions (DMPs) and regions (DMRs) across genomic features. Stacked percent bar graphs of the percent overlap of significant ancestry-related DMPs and DMRs with various genomic features (*green*) and detailed chromatin states (*blue*).

Abbreviations: *IGR*, intergenic region; *TSS*, transcription start site; *UTR*: untranslated region.

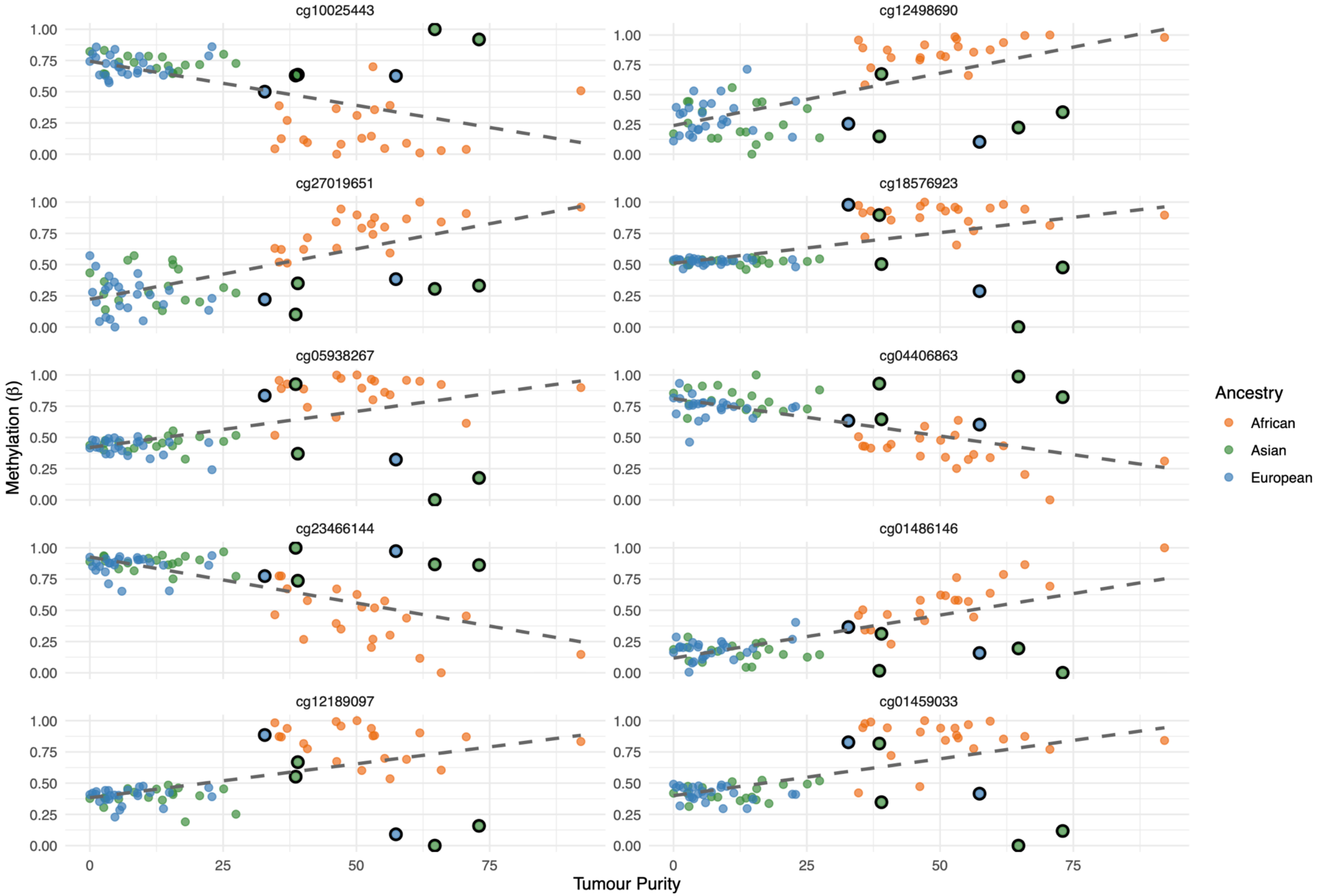
cg10025443
cg12498690
cg27019651
cg18576923
cg05938267
cg04406863
cg23466144
cg01486146
cg12189097
cg01459033
Methylation (β)
Tumour Purity
0.00
0.25
0.50
0.75
1.00
0
25
50
75
Ancestry
African
Asian
European

108

Appendix Figure S7 - Methylation at top 10 ancestry-associated DMPs by tumour purity and ancestry. Scatterplots display β-values at the top 10 differentially methylated positions (DMPs) between African and non-African prostate tumours, plotted against tumour purity (T-luminal). Points are coloured by ancestry (African, European, Asian), and non-African samples within the highest tumour purity quartile (Q4) are outlined in black to highlight cross-ancestry comparability at matched purity. Dashed lines represent linear regression fits. Vertical separation of ancestries at similar purity levels suggests methylation differences are not solely driven by tumour composition but reflect ancestry-associated epigenetic differences. Probe IDs are shown per panel, ranked by significance (adjusted *p*-value).

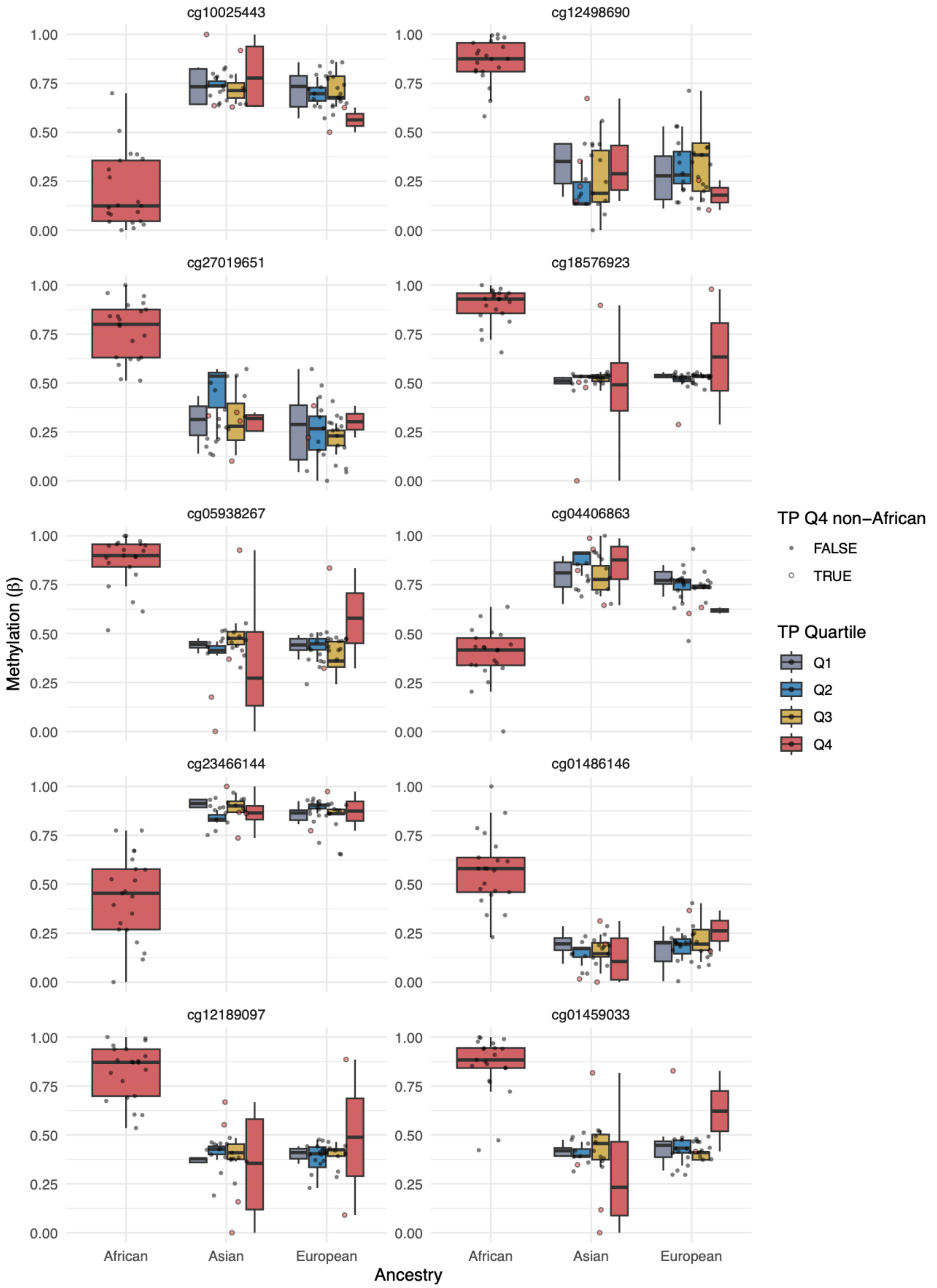

Appendix Figure S8 - Methylation at top 10 DMPs across tumour purity quartiles by ancestry. Boxplots show methylation (β-values) at the top 10 ancestry-associated differentially methylated positions (DMPs) stratified by ancestry (African, European, Asian) and tumour purity (TP) quartile (Q1-Q4). Jittered points represent individual tumour samples.

Non-African samples within the highest tumour purity quartile (Q4) are further distinguished by point outline and shading. For several DMPs, methylation differences between ancestries persist across multiple purity strata, supporting an ancestry-associated signal. Panel titles indicate DMP identifiers, ordered by adjusted *p*-value.

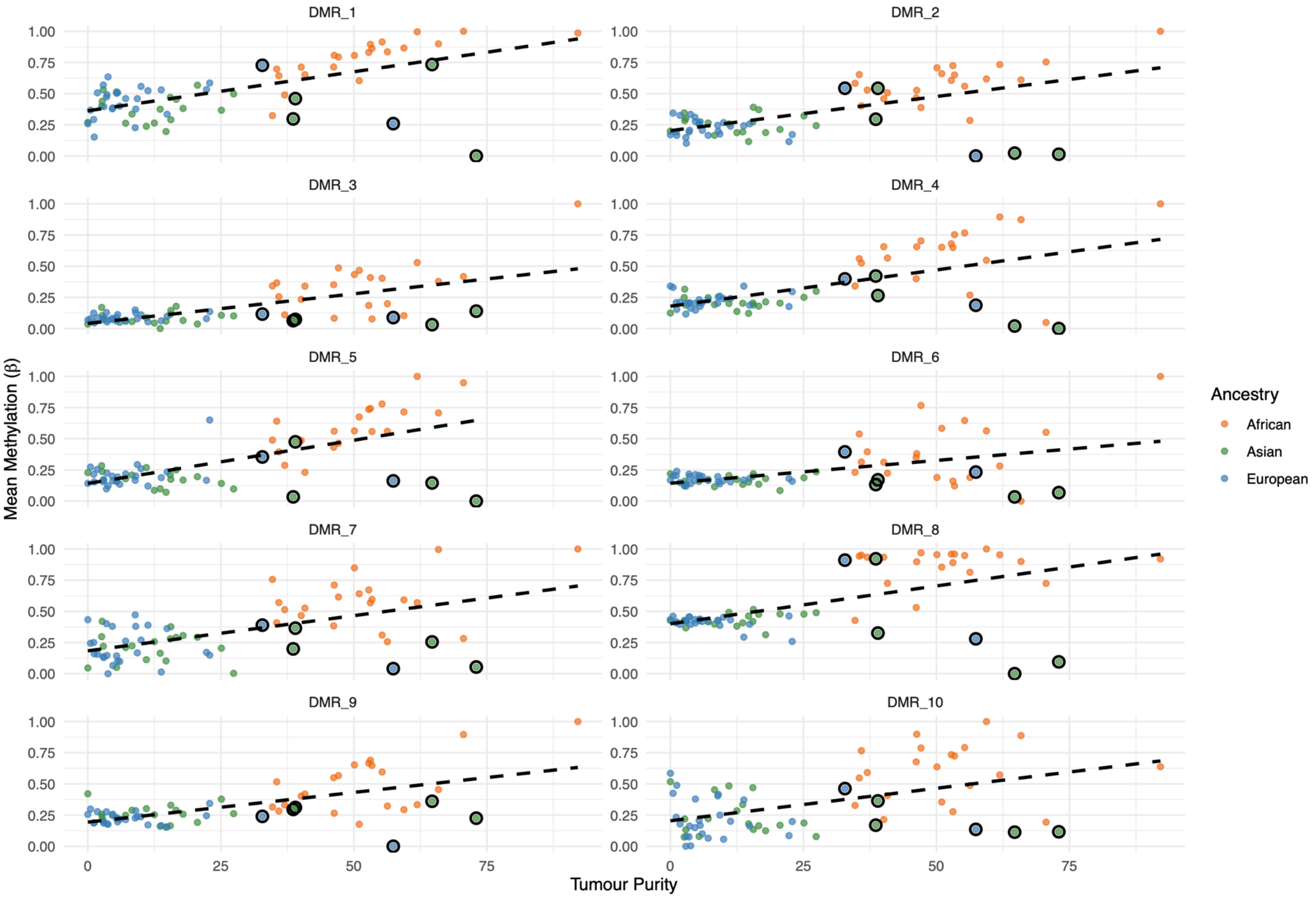
DMR_1
DMR_2
DMR_3
DMR_4
DMR_5
DMR_6
DMR_7
DMR_8
DMR_9
DMR_10
Mean Methylation (β)
Tumour Purity
0.00
0.25
0.50
0.75
1.00
0
25
50
75
Ancestry
African
Asian
European

Appendix Figure S9 - Methylation at top 10 ancestry-associated DMRs by tumour purity and ancestry. Scatterplots illustrate mean methylation levels (β) across the top 10 differentially methylated regions (DMRs) between African and non-African prostate tumours, plotted against tumour purity (T-luminal). Colours indicate ancestry (African, European, Asian), and non-African samples within the highest tumour purity quartile (Q4) are outlined in black. Regression lines are shown per DMR. Where ancestral groups remain distinct across purity levels, results suggest ancestry-specific regional methylation not explained by cell composition alone.

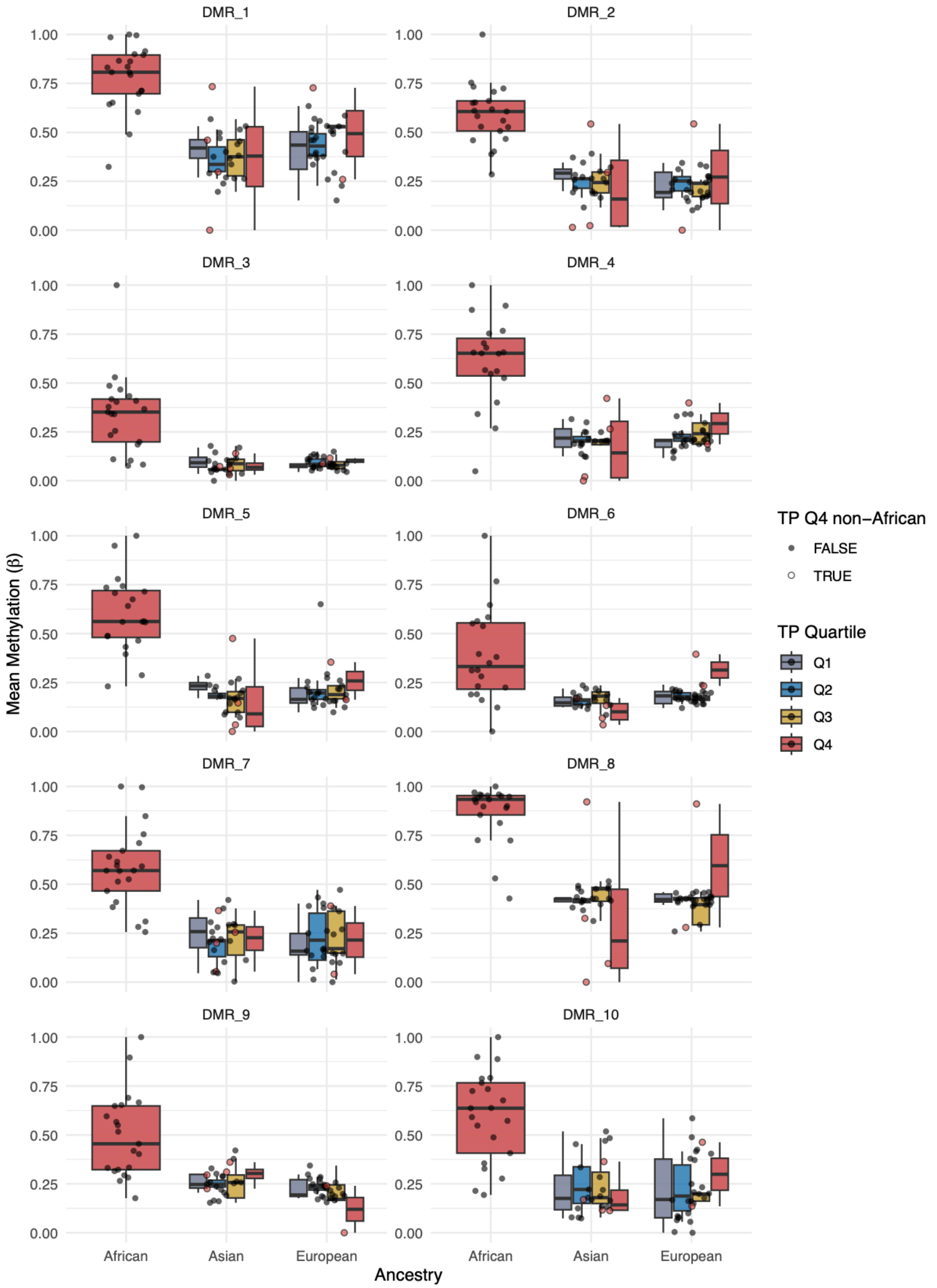

Appendix Figure S10 - Methylation at top 10 DMRs across tumour purity quartiles by ancestry. Boxplots show mean methylation levels at the top 10 ancestry-associated DMRs, stratified by ancestry and tumour purity (TP) quartile. Ancestry-specific methylation differences that persist across tumour purity quartiles indicate robustness to compositional variation.

Jittered points denote individual tumours, and non-African samples within the highest tumour purity quartile (Q4) are outlined and shaded.

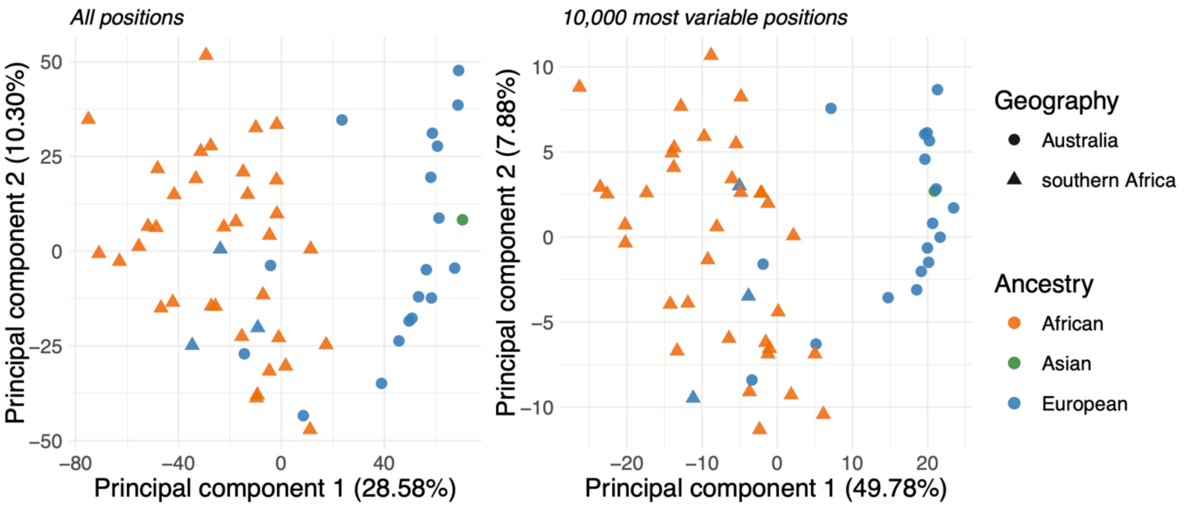

Appendix Figure S11 - Principal component analysis (PCA) of African *versus* non-African prostate tumours in the ancestry-associated validation cohort. PCA plot of 57 EPICv2 samples across (*left*) all positions and (*right*) the 10,000 most variable positions. Samples are annotated according to geography: Australia (*n* = 19) and southern Africa (*n* = 38); and ancestry: African (*n* = 35), Asian (*n* = 1) and European (*n* = 21). Seven of the African tumours include those reclassified as “presumably PCa”.

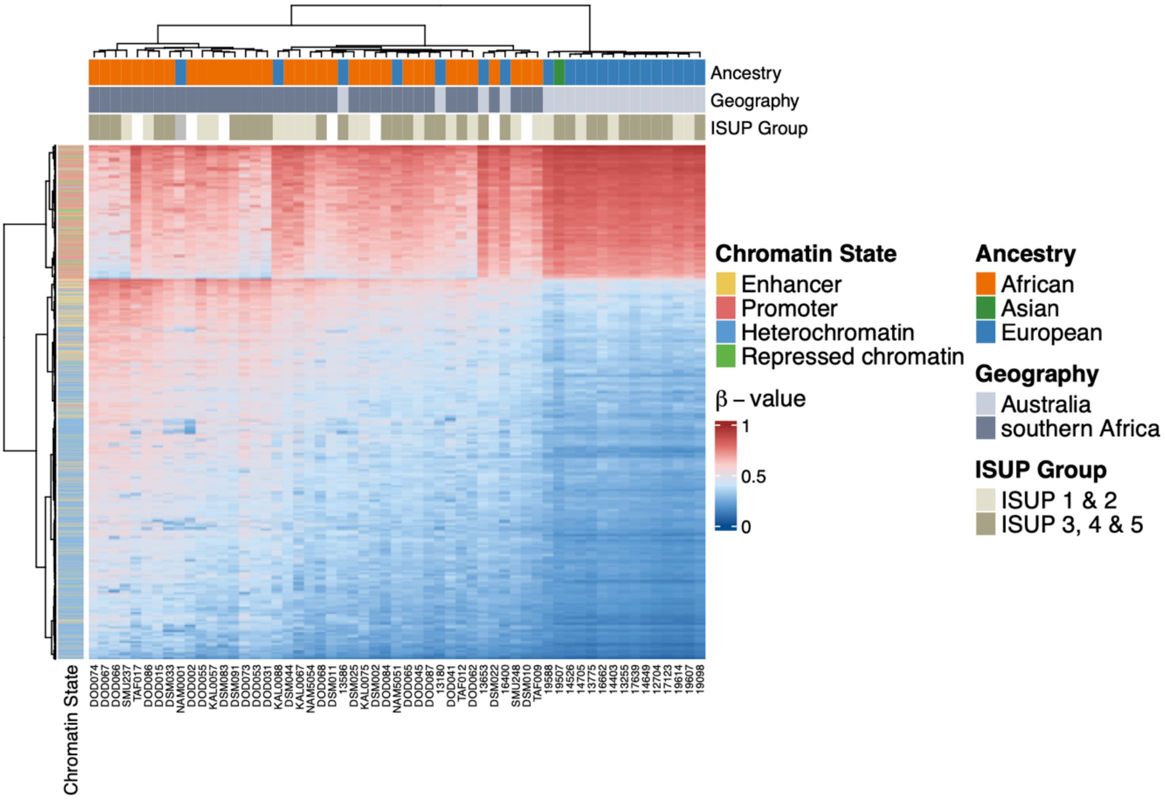

Appendix Figure S12 - DMP cluster analysis heatmap by ancestry, geography, ISUP grade group and chromatin state in the ancestry-associated validation cohort. Cluster analysis heatmap of the top 5,000 (of 14,160 total) DMPs identified between African and non-African prostate tumour-derived samples in the validation cohort. Rows correspond to CpG sites (n = 5,000) and columns, to individuals (n = 57). The methylation level is represented by a β-value between 0 and 1, with 0 being completely unmethylated (blue) and 1, fully methylated (red). Notably, seven of the African tumours include those reclassified as "presumably PCa".

Abbreviations: *DMP*, differentially methylated position; *ISUP*, International Society of Urological Pathology.

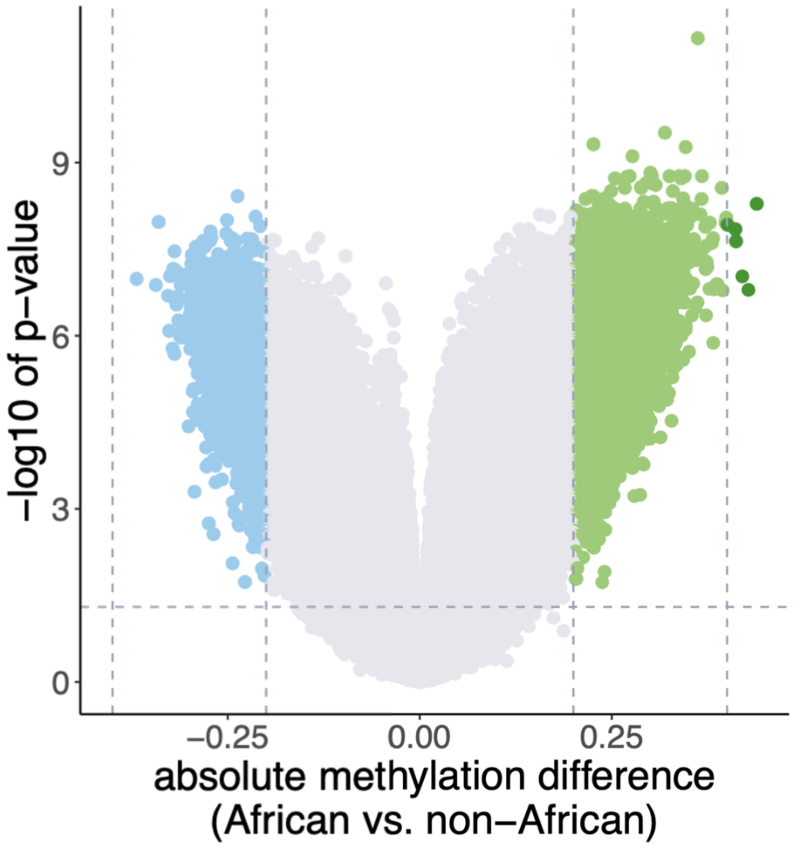

Appendix Figure S13 - Volcano plot for differentially methylated positions (DMPs) between African and non-African prostate tumour-derived samples in the ancestry-associated validation cohort. Grey points represent non-significant DMPs, while green and blue points represent hypermethylated and hypomethylated DMPs in Africans *versus* non-Africans, respectively. Notably, seven of the African tumours include those reclassified as “presumably PCa”. Dashed lines indicate cutoffs for significance (BH FDR p <0.05, $|\Delta\beta| \geq 20\%$ and 40%).

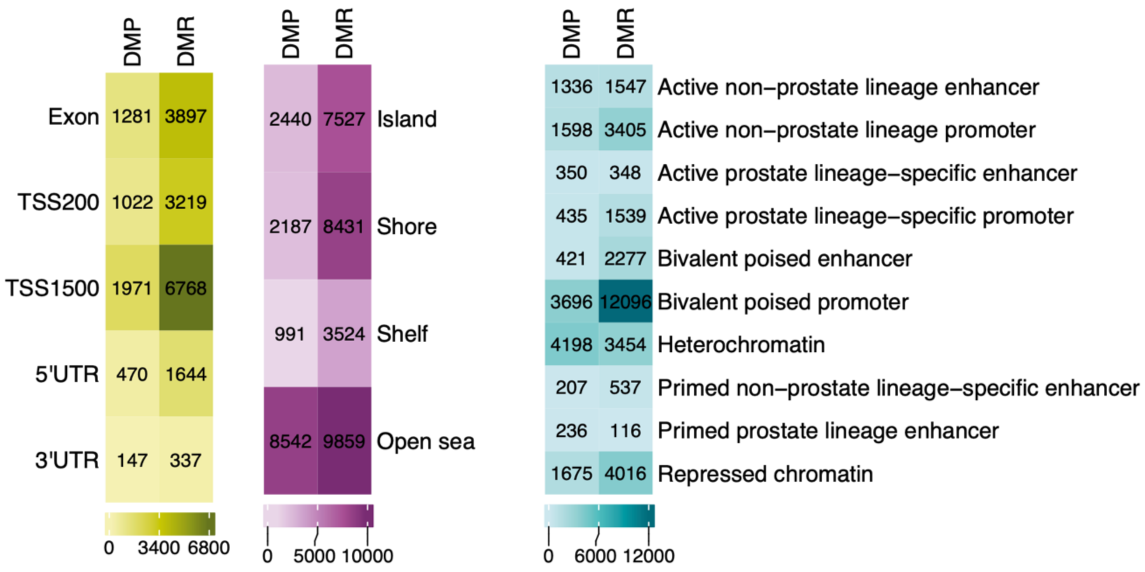

Appendix Figure S14 - Ancestry-associated DMP and DMR enrichment across gene and CpG island regions, and chromatin state contexts in the validation cohort. Heatmap showing (*green*) gene region enrichment, (*purple*) CpG island-related region enrichment, and (*blue*) prostate-specific chromatin state enrichment of ancestry-associated DMP and DMR-related CpG sites across various contexts in the validation cohort.

Abbreviations: *DMP*, differentially methylated position; *DMR*, differentially methylated region; *TSS*, transcription start site; *UTR*, untranslated region.

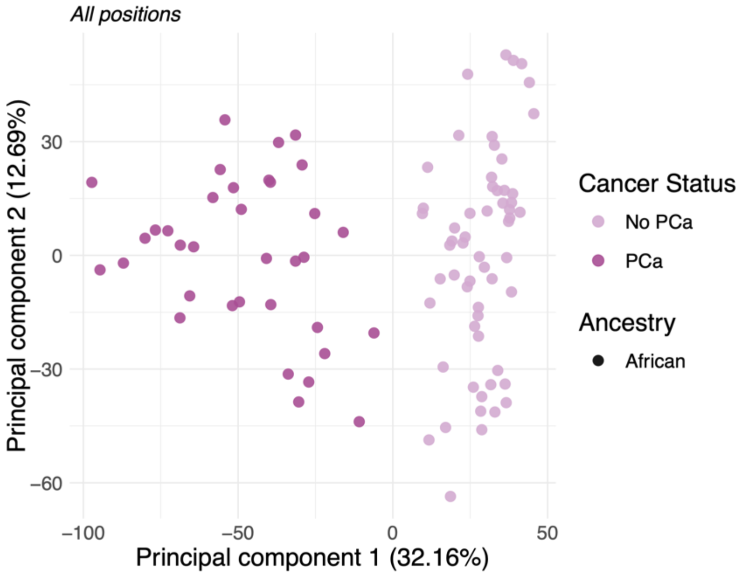

Appendix Figure S15 - Principal component analysis (PCA) of African prostate tumour *versus* normal tissue. PCA plot of 93 African EPICv2 samples across all CpG positions. African samples are annotated according to cancer status: those with PCa (n = 35, including 7 “presumably PCa”), and those without PCa (n = 58).

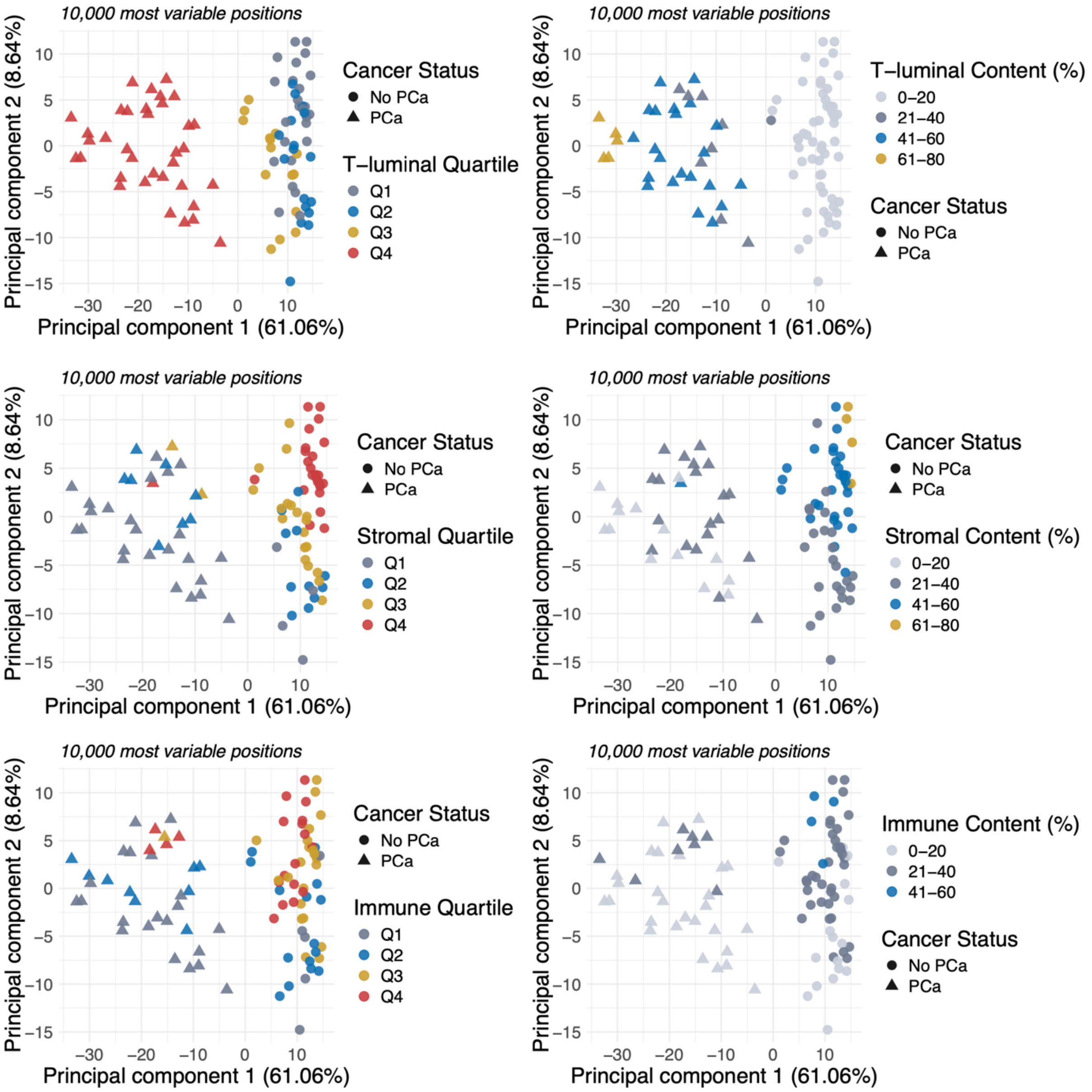

Appendix Figure S16 - Principal component analysis (PCA) of African prostate tumour *versus* normal tissue by cell types. PCA plot of 93 African EPICv2 samples across the 10,000 most variable positions indicating cancer status i.e. those with PCa (n = 35, including 7 "presumably PCa"), and those without PCa (n = 58). Samples are further annotated according to T-luminal quartile: Q1 (n = 26), Q2 (n = 17), Q3 (n = 15) and Q4 (n = 35); estimated T-luminal percentage: 0-20 (n = 57), 21-40 (n = 9), 41-60 (n = 22) and 61-80 (n = 5); stromal cell quartile: Q1 (n = 28), Q2 (n = 19), Q3 (n = 21) and Q4 (n = 25); estimated stromal percentage: 0-20 (n = 14), 21-40 (n = 44), 41-60 (n = 31) and 61-80 (n = 4); immune cell quartile: Q1 (n = 26), Q2 (n = 24), Q3 (n = 23) and Q4 (n = 20); estimated immune cell percentage: 0-20 (n = 43), 21-40 (n = 46) and 41-60 (n = 4).

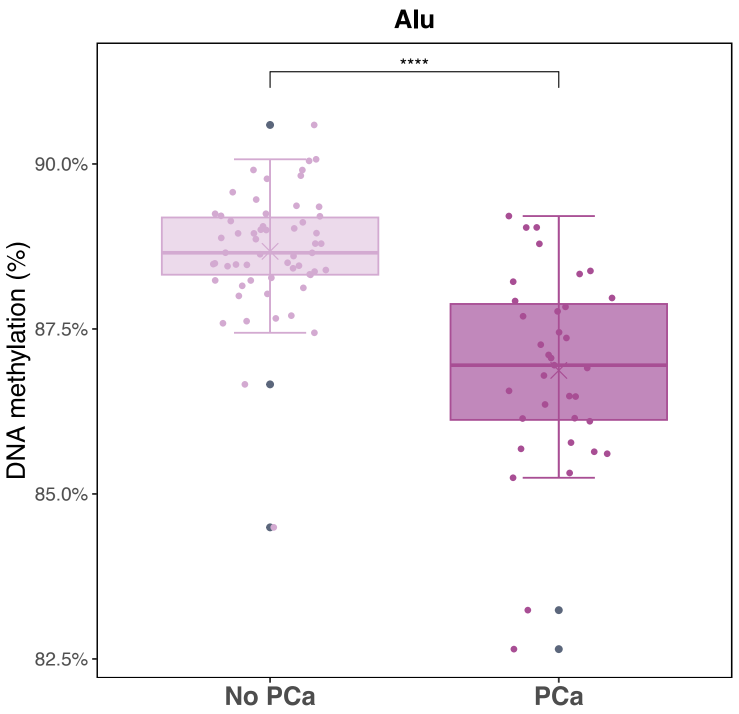

Appendix Figure S17 - Global DNA methylation across Alu repetitive elements in African prostate tumour *versus* normal tissue. Boxplot of global DNA methylation levels (covariate adjusted) in African prostate tumour (including 7 “presumably PCa”) and non-tumour samples across Alu repetitive elements. Each dot represents the median methylation value for each patient. The “X” indicates group mean.

Abbreviations: ****, $p \leq 0.0001$.

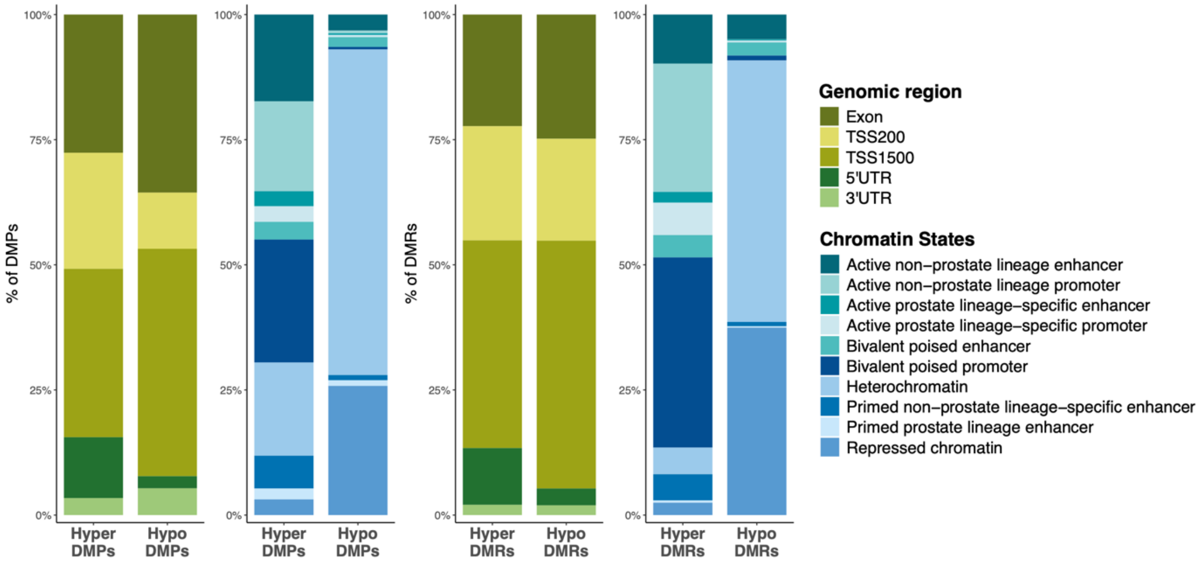

Appendix Figure S18 - Tumour-associated hyper- and hypomethylated differentially methylated positions (DMPs) and regions (DMRs) across genomic features and detailed chromatin states. Stacked percent bar graphs of the percent overlap of significant tumour-associated DMPs and DMRs across (*green*) various genomic features and (*blue*) detailed chromatin states.

Abbreviations: *TSS*, transcription start site; *UTR*: untranslated region.

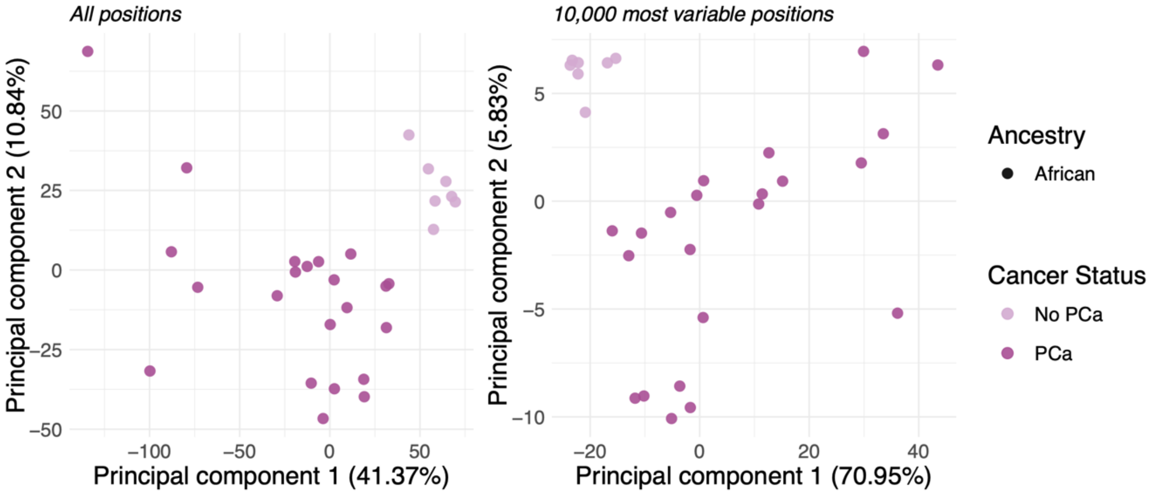

Appendix Figure S19 - Principal component analysis (PCA) of African prostate tumour *versus* normal tissue in the tumour-associated validation cohort. PCA plot of 29 African EPICv1 samples across (*left*) all positions and (*right*) the 10,000 most variable positions. African samples are annotated according to cancer status: those with PCa ($n = 22$), and those without PCa ($n = 7$).

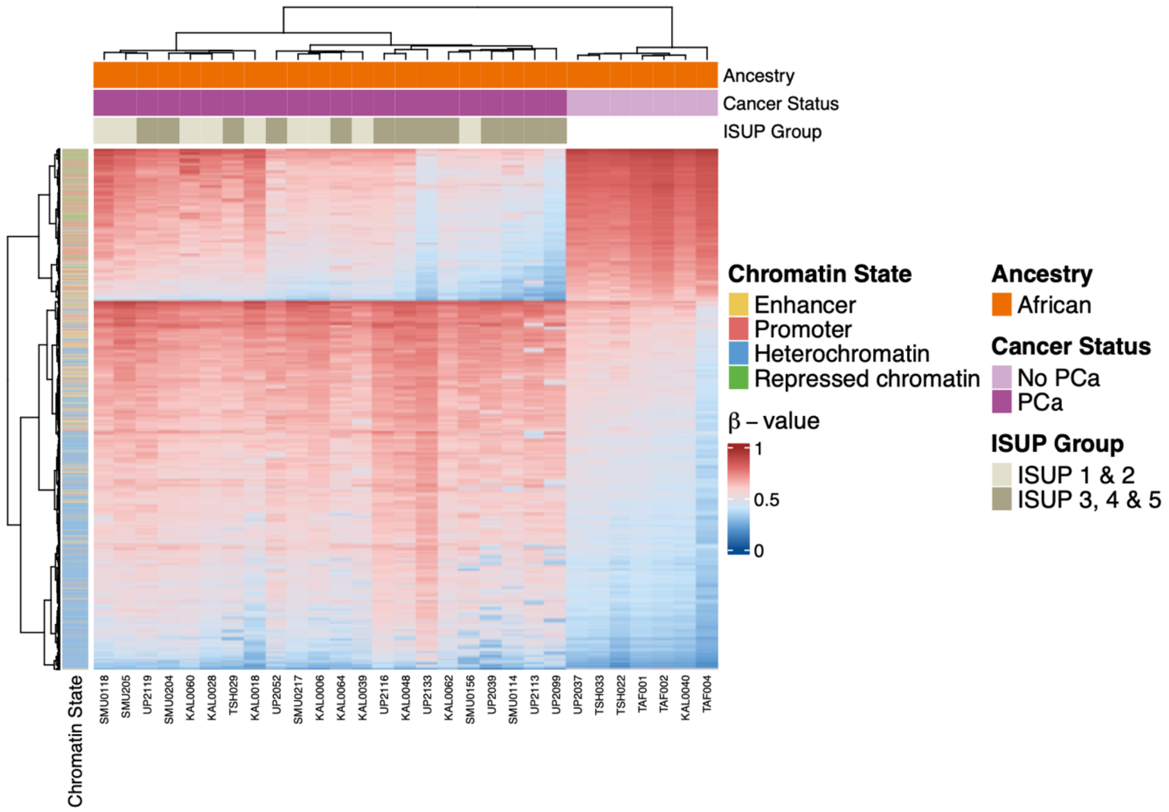

Appendix Figure S20 - DMP cluster analysis heatmap by cancer status, ISUP grade group and chromatin state in the tumour-associated validation cohort. Cluster analysis heatmap of the 5,520 significant DMPs identified between African prostate tumour and non-tumour samples in the validation cohort. Rows correspond to CpG sites (n = 5,520) and columns, to individuals (n = 29). The methylation level is represented by a β-value between 0 and 1, with 0 being completely unmethylated (blue) and 1, fully methylated (red). Samples are annotated according to cancer status and ISUP grade group.

Abbreviations: *DMP*, differentially methylated position; *ISUP*, International Society of Urological Pathology.

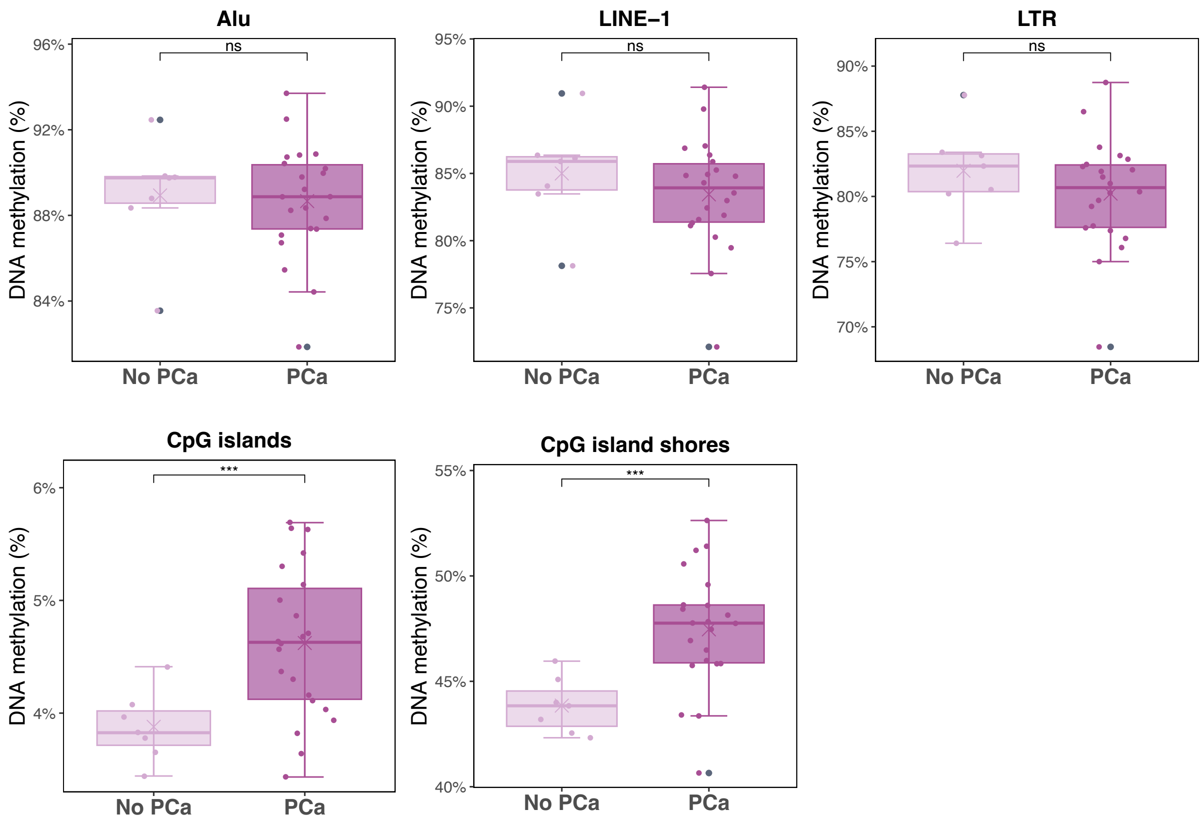

Appendix Figure S21 - Global DNA methylation across repetitive elements and CpG island-related regions in African prostate tumour *versus* normal tissue in the tumour-associated validation cohort. In the validation cohort, boxplots of global DNA methylation levels (covariate adjusted) in African prostate tumour and non-tumour samples across Alu repetitive elements, LINE-1 repetitive elements and long tandem repeats (LTRs), as well as across CpG islands and CpG island shores. Each dot represents the median methylation value for each patient. The "X" indicates group mean.

Abbreviations: *ns*, not significant; ***, $p \leq 0.001$.

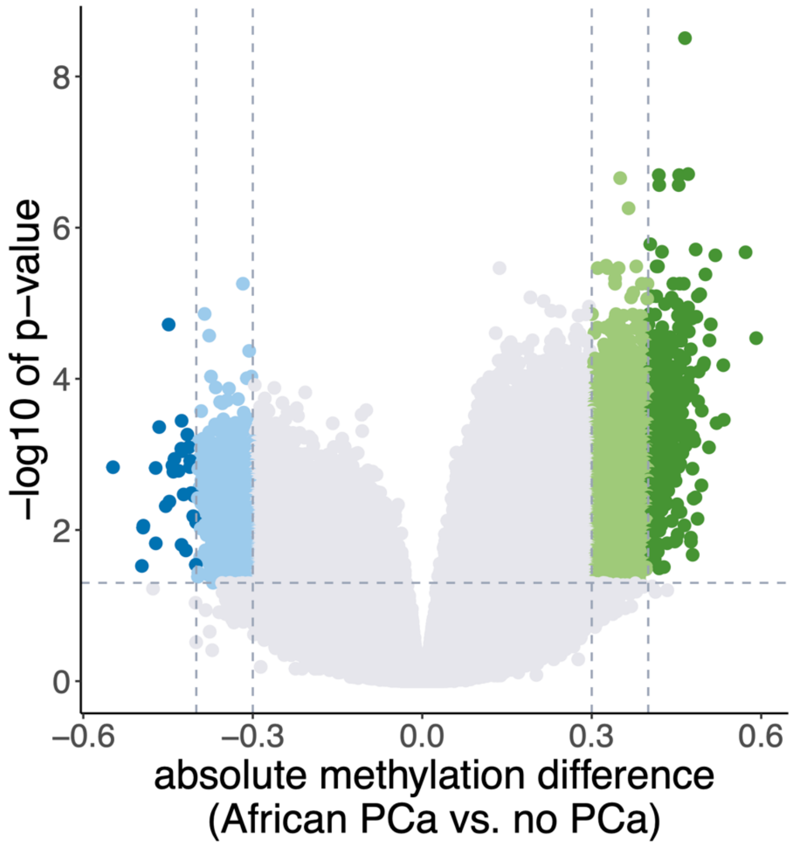

Appendix Figure S22 - Volcano plot for differentially methylated positions (DMPs) between African prostate tumour and normal tissue in the tumour-associated validation cohort. Grey points represent non-significant DMPs, while green and blue points represent hypermethylated and hypomethylated DMPs in African prostate tumour *versus* normal, respectively. Dashed lines indicate cutoffs for significance (BH FDR p <0.05, $|\Delta\beta| \geq 30\%$ and 40%).

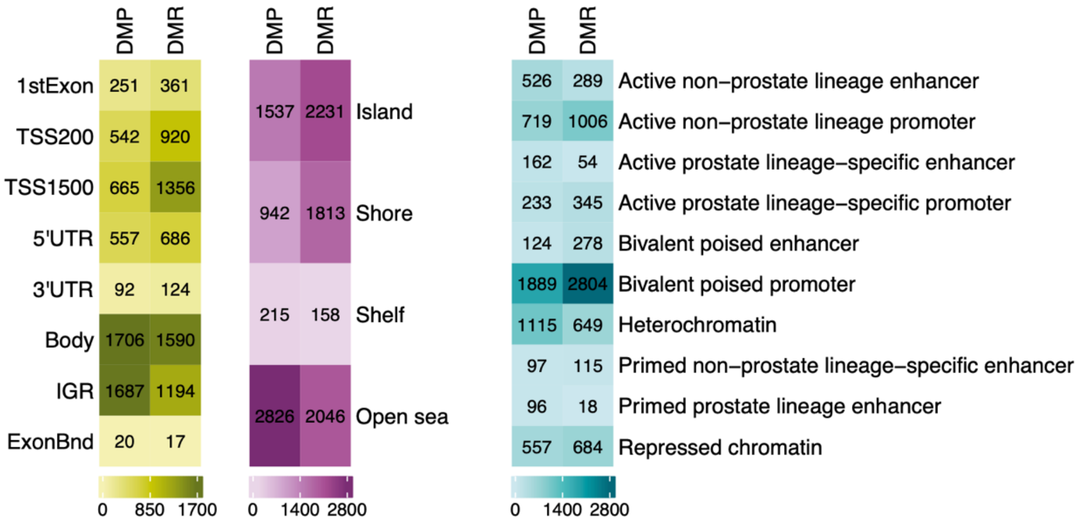

Appendix Figure S23 - Tumour-associated DMP and DMR enrichment across gene and CpG island regions, and chromatin state contexts in the validation cohort. Heatmap showing (*green*) gene region enrichment, (*purple*) CpG island-related region enrichment, and (*blue*) prostate-specific chromatin state enrichment of ancestry-associated DMP and DMR-related CpG sites across various contexts in the validation cohort. Abbreviations: *DMP*, differentially methylated position; *DMR*, differentially methylated region; *ExonBnd*, within 20 bases of an exon boundary (i.e. the start or end of an exon); *TSS*, transcription start site; *UTR*, untranslated region.
