# Supplementary material for: Methylation reprogramming associated with aggressive prostate cancer and ancestral disparities
Source: Mol Syst Biol. 2025 Oct 7;21(12):1676–701. doi: 10.1038/s44320-025-00153-x (PMC12673094; doi:10.1038/s44320-025-00153-x)
Supplement: Supplementary file 44 — Expanded View Figures [file 44320_2025_153_MOESM44_ESM.pdf]

Expanded View Figures

Figure EV1. Identifying EPICv1 and EPICv2 probes that overlap southern African polymorphic variants.

Workflow for the identification of EPIC probes (v1.0 and v2.0) overlapping African SNV and indel variants, rendering filtering resources. Consensus probe filtering resources are based on germline variants (MAF > 0.01) from 99 southern African men. indel insertion and deletion, MAF minor allele frequency, SBE single base extension, SNV single-nucleotide variant, VCF variant call format.

© The Author(s)

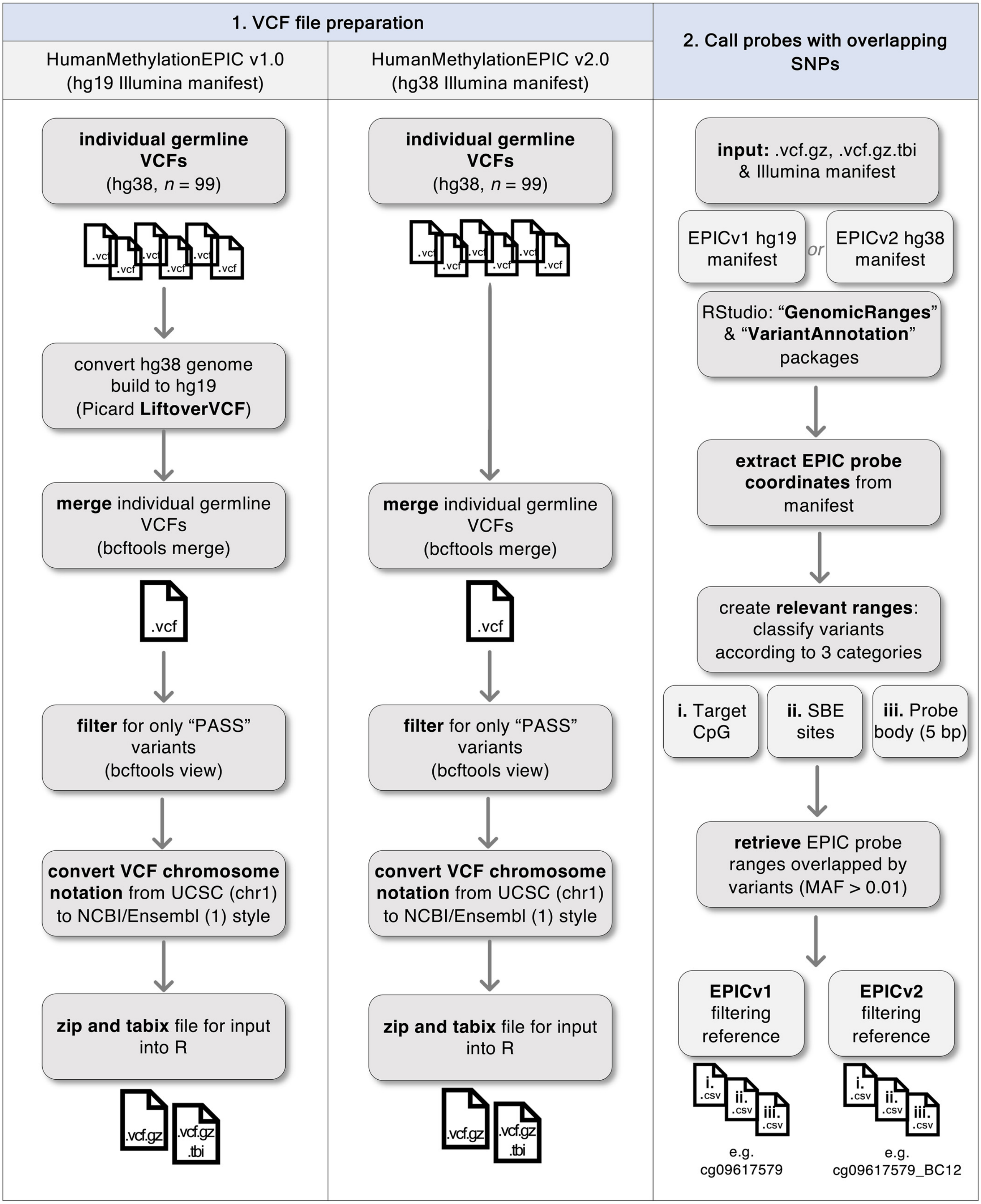

1. VCF file preparation
HumanMethylationEPIC v1.0 (hg19 Illumina manifest)
HumanMethylationEPIC v2.0 (hg38 Illumina manifest)
2. Call probes with overlapping SNPs
individual germline VCFs (hg38, n = 99)
.vcf
convert hg38 genome build to hg19 (Picard LiftoverVCF)
merge individual germline VCFs (bcftools merge)
filter for only "PASS" variants (bcftools view)
convert VCF chromosome notation from UCSC (chr1) to NCBI/Ensembl (1) style
zip and tabix file for input into R
.vcf.gz
.vcf.gz .tbi
input: .vcf.gz, .vcf.gz.tbi & Illumina manifest
EPICv1 hg19 manifest
or
EPICv2 hg38 manifest
RStudio: "GenomicRanges" & "VariantAnnotation" packages
extract EPIC probe coordinates from manifest
create relevant ranges: classify variants according to 3 categories
i. Target CpG
ii. SBE sites
iii. Probe body (5 bp)
retrieve EPIC probe ranges overlapped by variants (MAF > 0.01)
EPICv1 filtering reference
EPICv2 filtering reference
i. .csv
ii. .csv
iii. .csv
e.g. cg09617579
e.g. cg09617579_BC12

 © The Author(s)

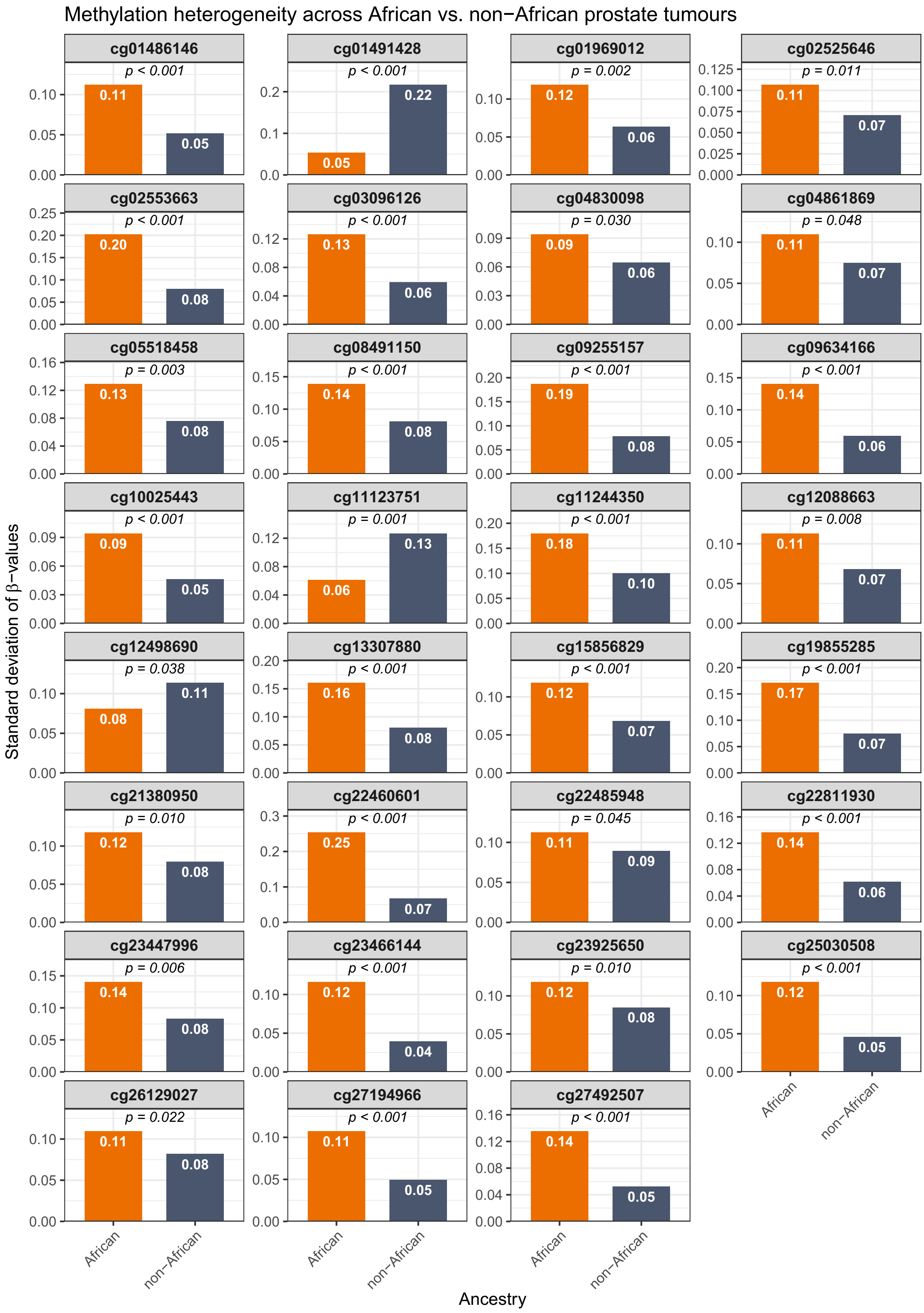
Methylation heterogeneity across African vs. non−African prostate tumours
cg01486146
p < 0.001
0.11
0.05
cg01491428
p < 0.001
0.05
0.22
cg01969012
p = 0.002
0.12
0.06
cg02525646
p = 0.011
0.11
0.07
cg02553663
p < 0.001
0.20
0.08
cg03096126
p < 0.001
0.13
0.06
cg04830098
p = 0.030
0.09
0.06
cg04861869
p = 0.048
0.11
0.07
cg05518458
p = 0.003
0.13
0.08
cg08491150
p < 0.001
0.14
0.08
cg09255157
p < 0.001
0.19
0.08
cg09634166
p < 0.001
0.14
0.06
cg10025443
p < 0.001
0.09
0.05
cg11123751
p = 0.001
0.06
0.13
cg11244350
p < 0.001
0.18
0.10
cg12088663
p = 0.008
0.11
0.07
cg12498690
p = 0.038
0.08
0.11
cg13307880
p < 0.001
0.16
0.08
cg15856829
p < 0.001
0.12
0.07
cg19855285
p < 0.001
0.17
0.07
cg21380950
p = 0.010
0.12
0.08
cg22460601
p < 0.001
0.25
0.07
cg22485948
p = 0.045
0.11
0.09
cg22811930
p < 0.001
0.14
0.06
cg23447996
p = 0.006
0.14
0.08
cg23466144
p < 0.001
0.12
0.04
cg23925650
p = 0.010
0.12
0.08
cg25030508
p < 0.001
0.12
0.05
cg26129027
p = 0.022
0.11
0.08
cg27194966
p < 0.001
0.11
0.05
cg27492507
p < 0.001
0.14
0.05
Standard deviation of β−values
African
non−African
Ancestry

◀ **Figure EV2. African prostate tumours exhibit elevated methylation variability at ancestry-associated DMPs.**

Standard deviation of DNA methylation β-values is shown for each ancestry group at the 31 top DMPs exhibiting significant variance differences by ancestry (Levene's test, $P < 0.05$). Each panel presents within-group variability across African and non-African tumours. In 90.3% of these DMPs, African tumours displayed the highest intra-group variability, supporting the presence of elevated ancestry-associated epigenetic heterogeneity.

 © The Author(s)

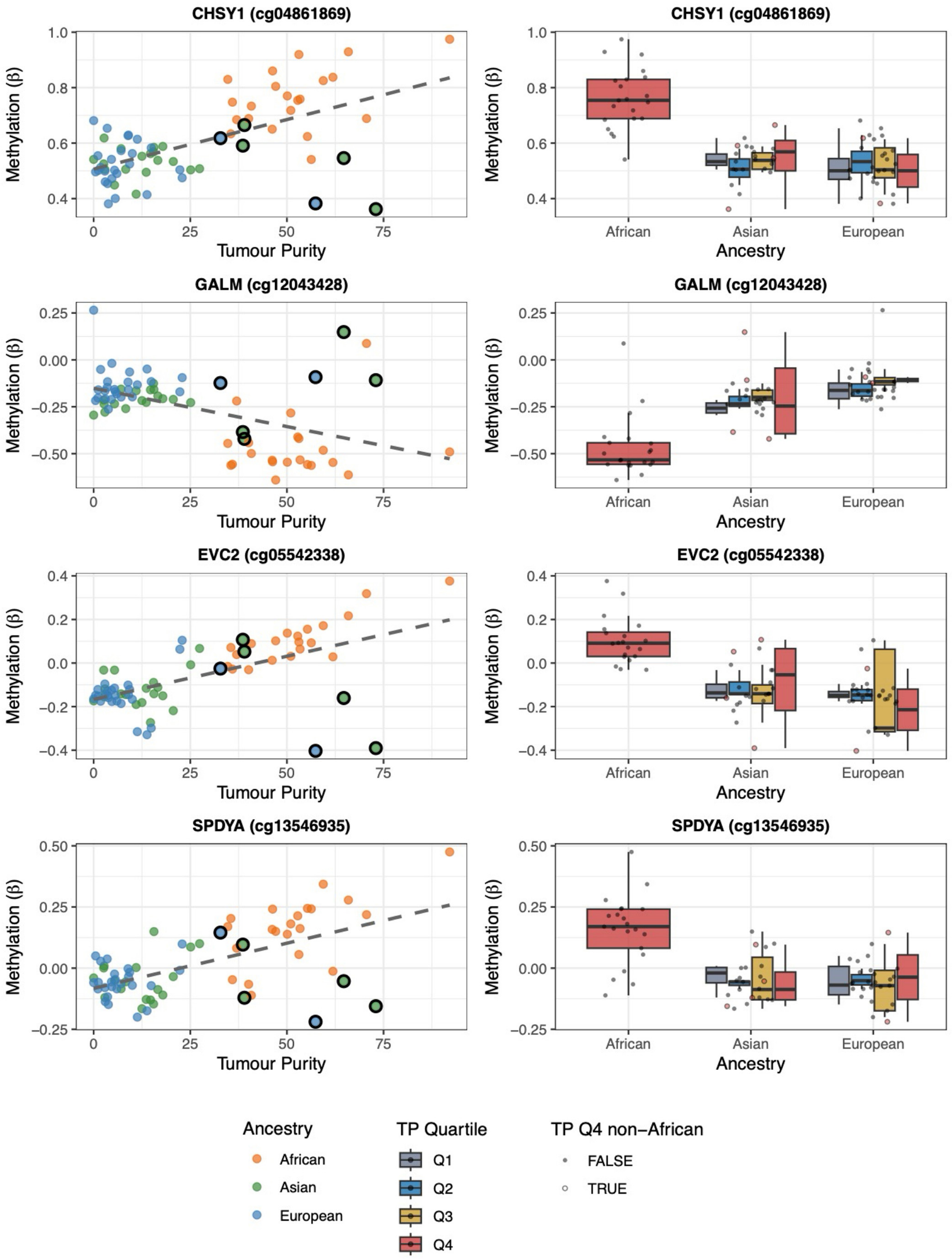
CHSY1 (cg04861869)
GALM (cg12043428)
EVC2 (cg05542338)
SPDYA (cg13546935)
Methylation (β)
Tumour Purity
Ancestry
African
Asian
European
TP Quartile
Q1
Q2
Q3
Q4
TP Q4 non−African
FALSE
TRUE

Figure EV3. Ancestry-associated methylation patterns at four key DMP genes and their relationship to tumour purity.

Scatterplots (left) and boxplots (right) depicting methylation levels at ancestry-associated CpG sites located in four genes of interest: *CHSY1*, *GALM*, *EVC2* and *SPDYA*. Scatterplots show methylation levels *versus* tumour purity across prostate tumours ($n = 70$), coloured by ancestry. Boxplots show ancestry-stratified methylation levels further grouped by tumour purity quartiles (Q1-Q4). Non-African Q4 tumour samples are outlined to highlight ancestry-related differences at high tumour purity.

 © The Author(s)
